# Bayesian phylogeny of sucrose transporters: ancient origins, differential expansion and convergent evolution in monocots and dicots

**DOI:** 10.3389/fpls.2014.00615

**Published:** 2014-11-12

**Authors:** Duo Peng, Xi Gu, Liang-Jiao Xue, James H. Leebens-Mack, Chung-Jui Tsai

**Affiliations:** ^1^Institute of Bioinformatics, University of GeorgiaAthens, GA, USA; ^2^Warnell School of Forestry and Natural Resources, University of GeorgiaAthens, GA, USA; ^3^Department of Genetics, University of GeorgiaAthens, GA, USA; ^4^Department of Plant Biology, University of GeorgiaAthens, GA, USA

**Keywords:** transporters, phloem, vascular evolution, angiosperms, molecular evolution, expression partitioning

## Abstract

Sucrose transporters (SUTs) are essential for the export and efficient movement of sucrose from source leaves to sink organs in plants. The angiosperm SUT family was previously classified into three or four distinct groups, Types I, II (subgroup IIB), and III, with dicot-specific Type I and monocot-specific Type IIB functioning in phloem loading. To shed light on the underlying drivers of SUT evolution, Bayesian phylogenetic inference was undertaken using 41 sequenced plant genomes, including seven basal lineages at key evolutionary junctures. Our analysis supports four phylogenetically and structurally distinct SUT subfamilies, originating from two *a*ncient *g*roups (AG1 and AG2) that diverged early during terrestrial colonization. In both AG1 and AG2, multiple intron acquisition events in the progenitor vascular plant established the gene structures of modern SUTs. Tonoplastic Type III and plasmalemmal Type II represent evolutionarily conserved descendants of AG1 and AG2, respectively. Type I and Type IIB were previously thought to evolve after the dicot-monocot split. We show, however, that divergence of Type I from Type III SUT predated basal angiosperms, likely associated with evolution of vascular cambium and phloem transport. Type I SUT was subsequently lost in monocots along with vascular cambium, and independent evolution of Type IIB coincided with modified monocot vasculature. Both Type I and Type IIB underwent lineage-specific expansion. In multiple unrelated taxa, the newly-derived *SUTs* exhibit biased expression in reproductive tissues, suggesting a functional link between phloem loading and reproductive fitness. Convergent evolution of Type I and Type IIB for SUT function in phloem loading and reproductive organs supports the idea that differential vascular development in dicots and monocots is a strong driver for SUT family evolution in angiosperms.

## Introduction

Sucrose is among the most abundant photoassimilates and the principal transport form of carbohydrates in many plants. The intracellular movement and long-distance transport of sucrose are mediated by sucrose transporters (SUTs), a group of transmembrane proteins belonging to the major facilitator superfamily (Sauer, 2007; Ayre, 2011). SUTs are proton-coupled symporters that transport sucrose either intercellularly across plasma membranes or intracellularly from the vacuole to the cytoplasm (reviewed in Kühn and Grof, 2010; Ayre, 2011). SUTs are encoded by small gene families in all plant species analyzed to date, from the primitive land plants *Physcomitrella* and *Selaginella* (Lalonde and Frommer, 2012; Reinders et al., 2012) to the woody perennial *Populus* (Payyavula et al., 2011). *SUT* genes are expressed throughout the plant body, with varying tissue- or cell-specificity depending on the isoform function. For instance, *SUT* genes involved in phloem loading have been localized to the companion cells and sieve elements (Riesmeier et al., 1993; Stadler et al., 1995; Truernit and Sauer, 1995), while those involved in sucrose uptake and other sink functions were preferentially expressed in tissues like pollen (Lemoine et al., 1999; Stadler et al., 1999), root (Flemetakis et al., 2003) or xylem (Decourteix et al., 2006). The various functions are also dictated by the differential (plasma or vacuolar) membrane localization of SUT proteins.

Several studies have reported on the phylogenetic organization of the plant *SUT* family, though with inconsistent and sometimes confusing nomenclature (Table 1). *SUT* genes were initially classified into three phylogenetic groups (Type I to Type III) based on experimentally characterized members and the full complement of the *SUT* families from *Arabidopsis* and *Oryza* (rice) (Aoki et al., 2003, 2012; Kühn, 2003; Lalonde et al., 2004; Shiratake, 2007; Zhou et al., 2007; Lalonde and Frommer, 2012). With the growing number of *SUT* genes identified, especially from the grasses, four distinct phylogenetic groups (Group 1 to Group 4) became evident, with Group 1 being monocot-specific (Sauer, 2007). This classification has been adopted in several subsequent studies (Braun and Slewinski, 2009; Payyavula et al., 2011), though again with inconsistent naming (Kühn and Grof, 2010; Doidy et al., 2012; Reinders et al., 2012). In two of the studies (Braun and Slewinski, 2009; Kühn and Grof, 2010), a fifth group was identified within the monocot-specific Group 1 (Table 1). For the sake of clarity, we have adhered to the nomenclature of Aoki et al. (2003) as amended by Reinders et al. (2012), with cross-referencing to the classification of Sauer (2007) (Table 1).

**Table 1:** **Summary of published SUT phylogenetic studies and subfamily classification**.

**References**	**SUT no.**	**Taxon no.^a^**	**SUT subfamilies**					**Tree construction method**
			**Ancient Group 1 (AG1)**		**Ancient Group 2 (AG2)**			
			**dicot**	**dicot/monocot**	**dicot/monocot**	**monocot**		
This study	233	41 (41)	Type I	Type III	Type II	Type IIB		Bayesian (MrBayes 3.2.1)
Doidy et al., 2012	88	22 (7)	SUT1	SUT4	SUT2 (IIA)	SUT2 (IIB)		Maximum parsimony (PAUP 4.0 β 10)
Reinders et al., 2012	70	35 (5)	Type I	Type III	Type IIA	Type IIB		Maximum likelihood (PhylML 3.0)
Aoki et al., 2012	92	38 (7)	Type I	Type III	Type II			Neighbor-joining
Lalonde and Frommer, 2012	99	35 (6)	SUT1	SUT4	SUT2			Maximum likelihood (MEGA 5.0)
Payyavula et al., 2011	74	19 (9)	Group 2	Group 4	Group 3	Group 1		Neighbor-joining (MEGA 4.1)
Kühn and Grof, 2010	41	16 (2)	SUT1	SUT4	SUT2	SUT3	SUT5	Maximum likelihood (PhylML 3.0)
Braun and Slewinski, 2009	42	16 (3)	Group 2	Group 4	Group 3	Group 1	Group 5	PHYLIP
Sauer, 2007	62	31 (3)	Group 2	Group 4	Group 3	Group 1		Clustal
Shiratake, 2007	43	20 (3)	SUC2/SUT1	SUC4	SUC3/SUT2			Neighbor-joining (ClustalW)
Zhou et al., 2007	57	26 (4)	Clade I	Clade II	Clade III			Maximum likelihood (Tree-Puzzle 5)
Lalonde et al., 2004	53	24 (5)	Clade I	Clade II	Clade III			Maximum parsimony (PAUP 4.0 β 10)
Kühn, 2003	44	22 (4)	SUT1	SUT4	SUT2			Maximum parsimony (PAUP 4.0 β 10)
Aoki et al., 2003	30	13 (3)	Type I	Type III	Type II			ClustalW

a *Values in parentheses denote the number of taxa for which the full complement of SUT (known at the time of this publication) was included*.

The eudicot-specific Type I (Group 2) is arguably the most extensively studied group, many of them have been localized to the plasma membrane and functionally associated with high-affinity sucrose uptake for phloem loading (reviewed in Sauer, 2007). The Type II group designated by Aoki et al. (2003) is split into two sub-groups in later studies (Sauer, 2007; Payyavula et al., 2011; Reinders et al., 2012). The canonical Type II (Type IIA or Group 3) contains both eudicot and monocot isoforms, whereas Type IIB (Group 1) represents the monocot-specific SUTs first reported by Sauer (2007). Several Type IIB members are plasma membrane-localized, high-affinity transporters (reviewed in Braun and Slewinski, 2009), a striking functional similarity to eudicot-specific Type I SUTs. Like Type IIB, several Type II members are also plasma membrane-localized (Barker et al., 2000; Meyer et al., 2000), but unlike Type IIB, Type II SUTs harbor an extended central cytoplasmic loop with unclear function in both eudicots and monocots (Aoki et al., 2003; Barth et al., 2003; Meyer et al., 2004; Hackel et al., 2006). Type III (Group 4) consists of all known tonoplast SUTs from both monocots and eudicots that are thought to facilitate sucrose release from vacuoles (Eom et al., 2011; Payyavula et al., 2011; Schneider et al., 2012). Dual (tonoplast and plasma membrane) localization has been reported for some Type III members (Chincinska et al., 2013). Phylogenetically distinct SUT orthologs are also found in lower vascular (*Selaginella*, spikemoss) and non-vascular (*Physcomitrella*, moss) plants (Lalonde and Frommer, 2012; Reinders et al., 2012), suggesting their divergence early during land plant evolution.

Given the essential roles of plasma membrane SUTs in apoplastic phloem loading (Gottwald et al., 2000; Scofield et al., 2002; Slewinski et al., 2009), the independent occurrence of two monophyletic SUT groups in eudicots (Type I) and monocots (Type IIB) with similar functions is intriguing. Because Type I is absent in monocots and lower plants, Reinders et al. (2012) proposed that this class evolved after the divergence of monocots and dicots. However, the taxonomic coverage lacked representation of basal angiosperm lineages to more properly infer the evolutionary history of SUT family in flowering plants. Here, we took advantage of the rapidly growing genomic resources to reconstruct a SUT phylogeny with 41 sequenced plants and 233 gene models using the Bayesian approach with the Markov chain Monte Carlo algorithm (Ronquist et al., 2012). In addition to algae, moss and spikemoss, we included *Phoenix dactylifera* (date palm, Al-Dous et al., 2011) and *Musa acuminate* (banana, D'Hont et al., 2012) that are basal to six genome-sequenced monocots, *Aquilegia coerulea* (Kramer, 2009) that is basal to 28 eudicots, and *Amborella trichopoda* that is basal to all sequenced angiosperms (Amborella Genome Project, 2013). This was instrumental for clarifying the evolutionary history of modern SUT families and their association with key developmental innovations in angiosperm evolution.

## Materials and methods

### Collection and curation of SUT sequences

SUT sequences from sequenced plant genomes were obtained from Phytozome v9.1, with the exceptions of *Amborella trichopoda* v1.0 (http://www.amborella.org), *Cicer arietinum* v1.0 (http://www.comparative-legumes.org), *Lotus japonicas* v2.5 (http://www.kazusa.or.jp/lotus), *Medicago truncatula* (Doidy et al., 2012 and cross-referenced with Phytozome v10), *Musa acuminate* v1 (http://banana-genome.cirad.fr) and *Phoenix dactylifera* v3 (http://qatar-weill.cornell.edu/research/datepalmGenome/download.html). The predicted gene models were subjected to manual curation to correct for gene structure prediction errors, such as miss-annotated splice junctions, guided by preliminary sequence alignment. Sequences that were too short (<300 amino acids) or represent possible splice variants were excluded from our analysis. SUT orthologs from *Galdieria sulphuraria* (red alga, http://genomics.msu.edu/galdieria), *Cyanidioschyzon merolae* (red alga, http://merolae.biol.s.u-tokyo.ac.jp), *Ostreococcus lucimarinus* and *Ostreococcus tauri* (green algae, http://genome.jgi-psf.org) were obtained using all higher plant SUT amino acid sequences as query to blast against the predicted protein databases of these species. Blast hits with >60% coverage of at least one query sequence was retained and manually inspected. This led to the identification of one homolog in *C. merolae*, five in *G. sulphuraria*, and two each in the green algae *O. lucimarinus* and *O. tauri*. No SUT homolog was found in *Chlamydomonas reinhardtii* or *Volvox carteri* green alga as reported previously (Reinders et al., 2012). Our pilot phylogenetic analysis showed that the green and red algal SUTs clustered together and joined all other plant SUTs in a single long branch, suggesting that algal SUTs can be used as outgroup. To reduce sequence divergence in multiple sequence alignment, only SUTs from red alga *G. sulphuraria* were included in subsequence analyses. The full list of 233 SUT gene models used in this study is provided in Supplemental Table 1. Exon-intron structures based on curated sequences of representative species were displayed using the Gene Structure Draw program (http://www.bioinformatics.uni-muenster.de/tools/strdraw).

### Sequence alignment and phylogenetic tree inference

Multiple sequence alignment was performed using MAFFT 7.037 (Katoh and Standley, 2013), with the “auto” option for alignment strategy selection. The resulting alignment was manually edited using alignment viewer in MEGA 5.2 (Tamura et al., 2011). Sites consisting of mostly gaps were removed. Pairwise sequence identity matrix was calculated by Clustal2.1 using default parameters. Bayesian inference of SUT phylogeny was performed using MrBayes v3.2.1 (Ronquist et al., 2012) executed on XSEDE (Extreme Science and Engineering Discovery Environment) through the CIPRES Science Gateway v3.3 (Miller et al., 2010). We performed pilot runs using mixed models to determine the best amino acid substitution models, and the “Jones” amino acid substitution model was chosen for subsequent runs. The likelihood model parameters were Rates = gamma, Ngammacat = 8, and Covarion = no. The Markov Chain Monte Carlo parameters were: Ngen = 12000000, nruns = 2, nchains = 8, Markov chain samplefreq = 200, and burn-in fraction = 0.5. Convergence was determined by 8750 post burn-in samples from two independent runs. The resulting phylogenetic reconstruction was converted to Newick format using Phylogeny.fr (Dereeper et al., 2008) and imported into MEGA5.2 for tree rendering. Maximum Likelihood analysis was also performed and returned similar results. We focus our discussion on the results from the Bayesian analyses since the Markov chain Monte Carlo search provides a statistically rigorous characterization of the distribution of plausible tree topologies, branch lengths and substitution model parameters given the sequence alignments (Holder and Lewis, 2003).

### Estimation of selection pressure

SUT nucleotide sequences were aligned using codon alignment option in MUSCLE implemented in MEGA 5.2. The resultant alignment was manually edited using alignment viewer in MEGA 5.2. Codon sites consisting of mostly gaps or with excessive diversity were removed. Maximum likelihood analysis of the ratio of non-synonymous and synonymous nucleotide substitution rates (ω = dN/dS) was conducted using PAML 4.5 (Yang, 2007). Modified branch site-model A (test of positive selection, model = 2, NS sites = 2) (Zhang et al., 2005) was used to test for positive selection for branches of interest from the SUT phylogeny. The likelihood was compared to a NULL model, in which foreground ω_2_ was fixed to 1. Likelihood ratio tests were conducted with df = 1. Bayes Empirical Bayes (BEB) analysis (Yang et al., 2005) was used to identify amino acid positions with high posterior possibility of being positively selected. To assess selection pressure of Type II and Type IIB SUTs, the edited alignment from above was divided into windows of 100 codons for dN and dS estimation using KaKs_Calculator 1.2 (Zhang et al., 2006). For *K*_s_ estimation of duplicated gene pairs, a custom perl script was written to batch-process *K*_s_ calculation using LWL method implemented in KaKs_Calculator 1.2 with coding sequence alignment results from MACSE v1.0.0.15 (Ranwez et al., 2011).

### Gene expression analysis

Affymetrix microarray data from vegetative and reproductive tissues of *O. sativa* L. ssp. *japonica* (Fujita et al., 2010; Kudo et al., 2013) and *Arabidopsis thaliana* (Schmid et al., 2005; Qin et al., 2009) were obtained from NCBI GEO (GSE14304, GSE41556, GSE19024, GSE29080, GSE5629-GSE5634, and GSE17343). The probe-set expression values were extracted by MAS5 using *affy* package from Bioconductor (Gautier et al., 2004). For soybean gene expression analysis, published RNA-Seq data (Hunt et al., 2011; Kim et al., 2011) and three seed datasets from the Goldberg laboratory (GSE29134, GSE29162 and GSE29163) were used. The single-end 75-bp reads were filtered to remove low-quality sequences using SolexaQA DynamicTrim (Cox et al., 2010) with default settings, and searched against the SILVA rRNA database (Quast et al., 2013), plant long non-coding RNA database (PLncDB, Jin et al., 2013), Rfam non-coding RNA database (Burge et al., 2013), and chloroplast (Saski et al., 2005) and mitochondrial genomes (Chang et al., 2013) to remove contaminants. The pre-processed reads were mapped to *Glycine max* v1.1 genome (Schmutz et al., 2010) using Tophat 2.0.10 (Trapnell et al., 2012). Only uniquely mapped reads were used to assess transcript abundance in reads per kilobase per million using cufflinks 2.1.1 (Trapnell et al., 2012). Expression values were normalized by Z-score transformation (Cheadle et al., 2003; Schmid et al., 2005) and visualized in heat maps using *pheatmap* package in *R*.

## Results

### Modern SUT gene family evolved from two ancient groups

**Figure 1:**
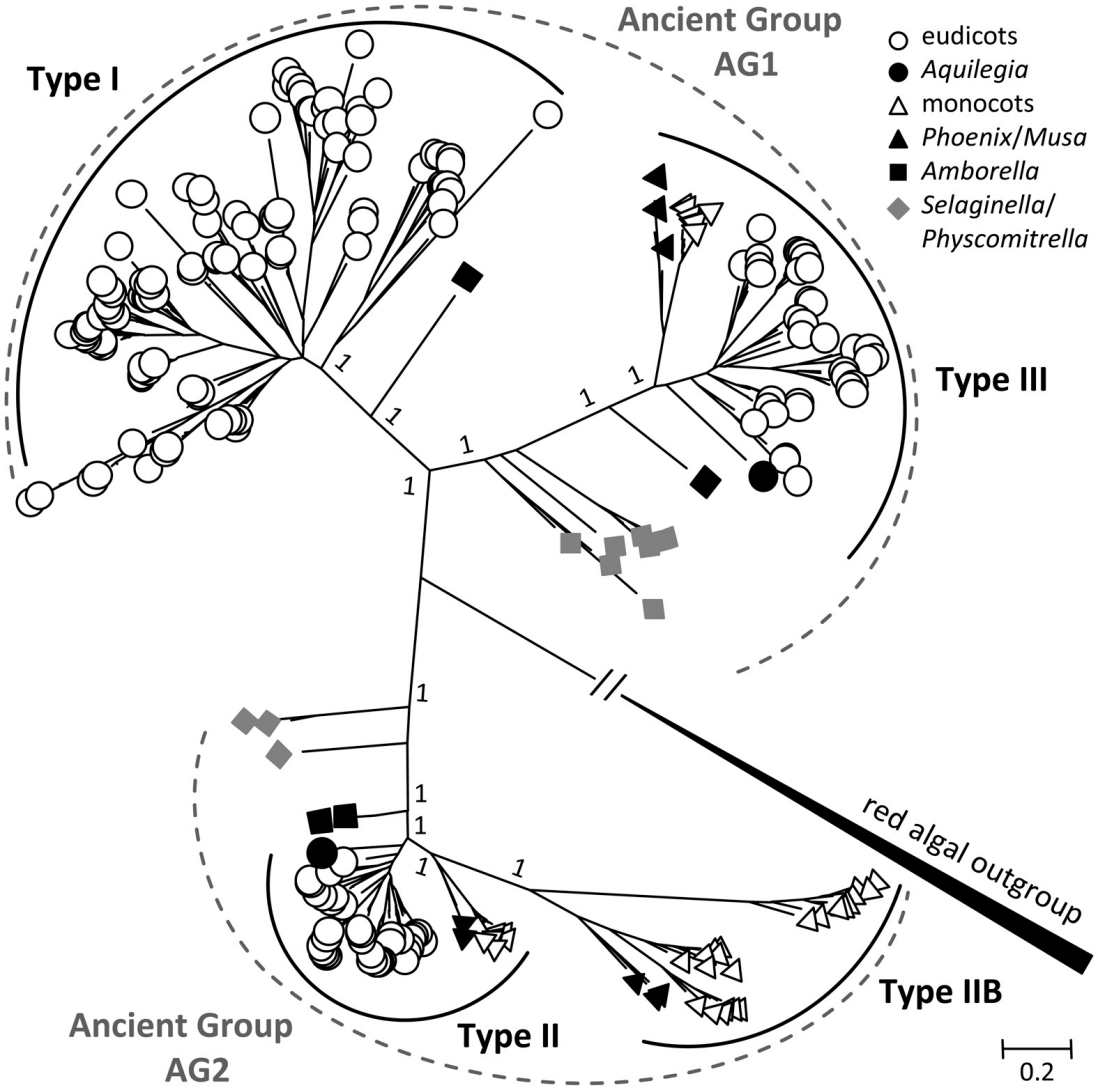
**Bayesian phylogenetic inference of SUT from 41 sequenced plant genomes**. Modern angiosperm SUTs form four distinct groups (Types I, II, IIB, and III) nested in two ancient groups AG1 and AG2, using red alga (*Galdieria sulphuraria*) SUTs as out group. Type II and Type III represent evolutionarily conserved groups with both monocot and eudicot members, whereas Type I and Type IIB are found only in eudicots and monocots, respectively. Basal lineages are denoted in solid symbols. Posterior probabilities for major nodes are shown.

The SUT phylogenetic tree inferred from the Bayesian method has strong support for the major nodes (Figure 1). The tree revealed two major clades for land plant SUTs, each with two previously recognized SUT groups—an overall topology similar to that reported elsewhere (Sauer, 2007; Braun and Slewinski, 2009; Payyavula et al., 2011; Doidy et al., 2012; Reinders et al., 2012). The smaller of the two clades consisted of Type II (Group 3) and Type IIB (Group 1), and the larger one comprised Type I (Group 2) and Type III (Group 4) (Table 1). The algal SUTs formed a strongly supported branch distinct from the two major clades (Figure 1), similar again in topology to that reported by Payyavula et al. (2011) and Reinders et al. (2012). The strong support for the two land plant clades descending from the algal clade suggests that modern plant *SUT* genes evolved from two different ancestors, hereafter referred to as *a*ncient *g*roups AG1 and AG2. SUT homologs from primitive plants (moss and spikemoss) were found in both AG1 and AG2, suggesting that the two ancient groups diverged early in the evolutionary history of land plants. To shed light on the subsequent evolution of AG1 and AG2 into the four modern SUT groups, we reconstructed two phylogenetic trees separately for the two major clades. This clade-specific approach was expected to reduce erroneous alignment among less closely related sequences, thereby improving the inference accuracy of SUT family evolution.

### AG1 duplication was lost in monocots but retained and expanded in eudicots

**Figure 2:**
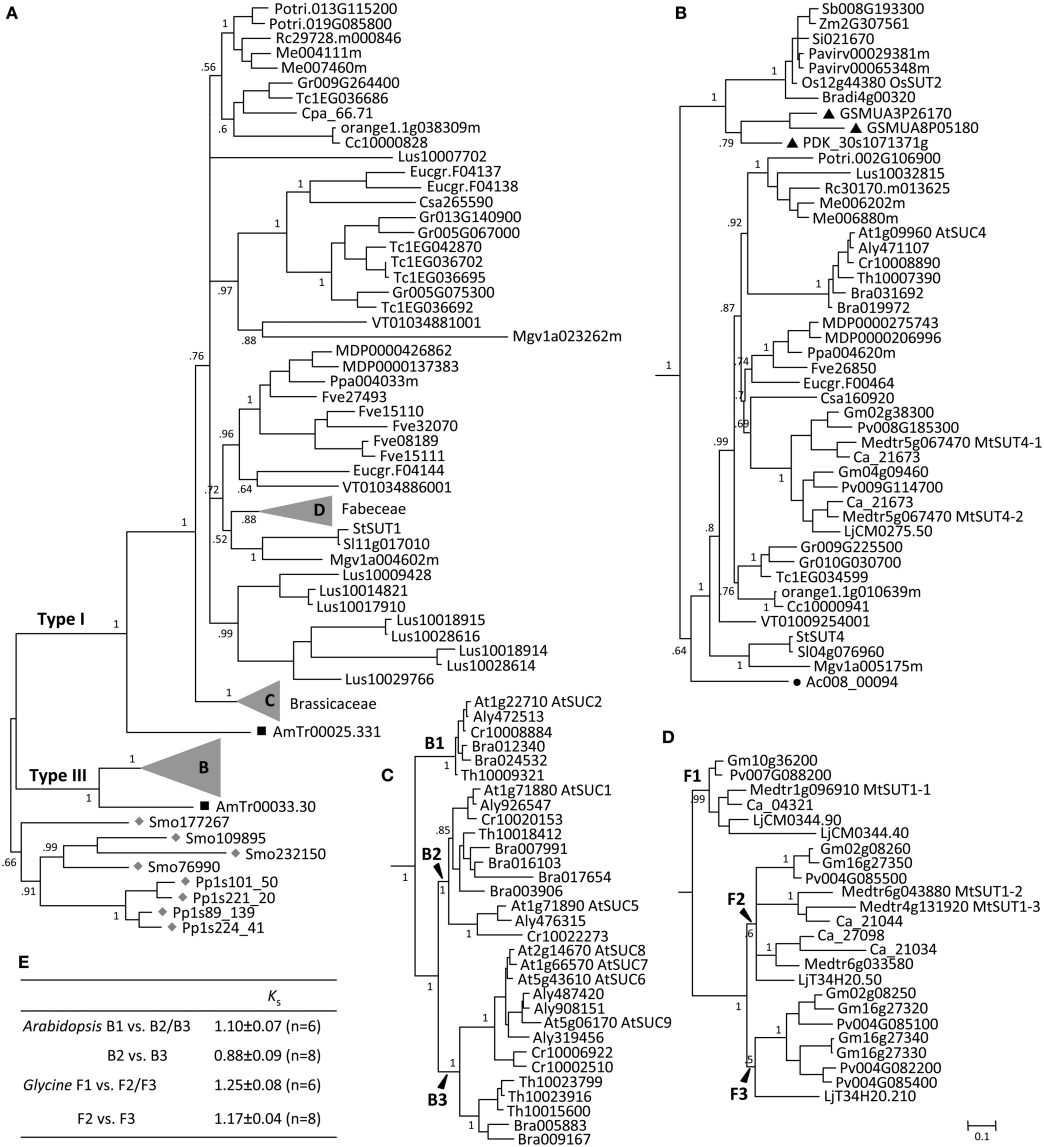
**Evolution of ancient group AG1 SUTs**. **(A)** Bayesian phylogenetic inference of AG1 SUTs supports the divergence of Type I and Type III form progenitor *Physcomitrella* and *Selaginella* SUTs (gray diamonds). *Amborella* members (black squares) are basal to modern angiosperm SUTs in both cases. **(B)** Zoom-in view of the angiosperm Type III branch. Eudicot and monocot members form distinct groups, with *Aquilegia* (black circle) and *Phoenix*/*Musa* (black triangles) occupying the respective basal positions. **(C)** Zoom-in view of Brassicaceae Type I branch with three subclades B1, B2, and B3. **(D)** Zoom-in view of the legume (Fabeceae) Type I branch with three subclades F1, F2, and F3. Posterior probabilities for major nodes are shown. Sequence names are provided in Supplemental Table 1. **(E)** *K*_s_ estimation between subcalde members of the expanded *Arabidopsis* and *Glycine* Type I SUTs. Values are means and standard deviations of all gene pairs within each comparison group.

**Figure 3:**
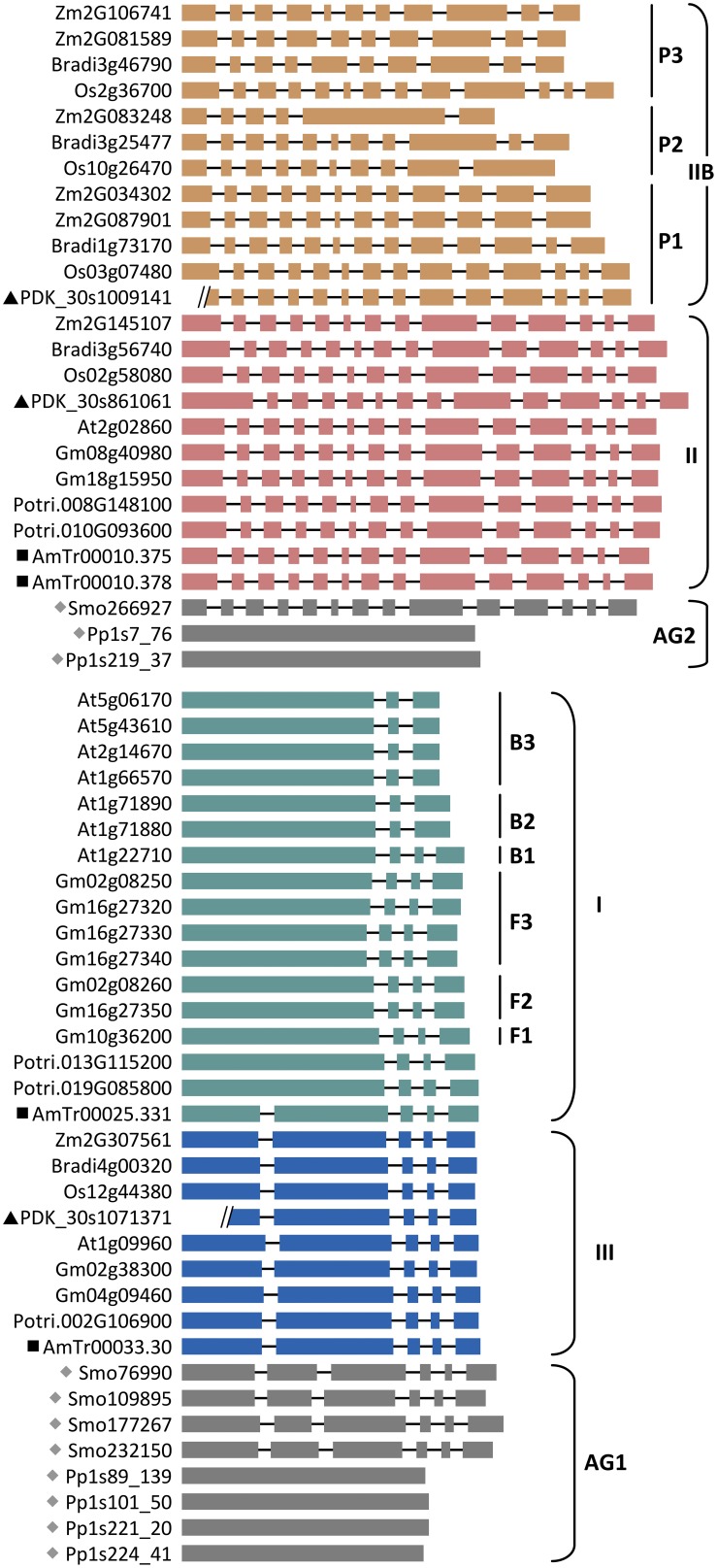
**Exon-intron structure of *SUT* genes in representative species arranged by SUT groups**. Ancient groups (AG1-AG2), Type I subclades of Brassicaceae (B1 to B3) and Fabeceae (F1 to F3), and Type IIB subclades of Poaceae (P1 to P3) are indicated. Basal lineages are denoted by solid symbols (gray diamonds, *Physcomitrella* (Pp) and *Selaginella* (Smo); black squares, *Amborella* (AmTr); black triangles, *Phoenix* (PDK). Introns are not drawn to scale, and two *Phoenix* gene models contain sequence gaps within the first exon. Other taxa are: *Arabidopsis* (At), *Brachypodium* (Bradi), *Glycine* (Gm), *Oryza* (Os), *Populus* (Potri), and *Zea* (Zm).

The AG1 clade is more than twice as large as the AG2 clade (Figure 1, Supplemental Table 1). Moss and spikemoss SUTs were basal to all other angiosperm SUTs, and both taxa have independently experienced *SUT* gene duplication, yielding four copies each in AG1 (Figure 2A). The angiosperm SUTs formed two strongly supported groups, corresponding to the eudicot-specific Type I and monocot- and eudicot-containing Type III (Figure 2A). In both cases, the basal position was occupied by an *Amborella* SUT homolog, suggesting that Type I and Type III SUTs diverged very early, perhaps before the advent of angiosperms. Both Type I and Type III *Amborella SUTs* have the same gene (exon-intron) structure that is also conserved in angiosperm Type III, but not Type I *SUTs* (Figure 3). This suggests that Type I was derived from Type III and has subsequently experienced an intron loss in the progenitor angiosperm. The complete absence of Type I SUTs in monocots supports the interpretation that the progenitor Type I SUT was lost from the ancestral monocot genome. Type I SUT was also absent in the current draft genome assembly of basal eudicot *Aquilegia*, although a partial match to Type I SUT was found on scaffold 6 flanked by repeat elements. This suggested either a genome assembly artifact or possibly an independent gene loss event in *Aquilegia*. Type III monocot and eudicot SUTs formed two distinct groups, with date palm/banana and *Aquilegia* members occupying the respective basal position (Figure 2B). Despite its broad taxonomic representation, the Type III clade is noticeably smaller than the eudicot-only Type I clade, mainly because the vast majority of angiosperm species sampled (71%) contain a single Type III gene (Figure 2B, Supplemental Table 1). This contrasts sharply with the eudicot-specific Type I clade where most (52%) taxa contain three or more copies (Figure 2A, Supplemental Table 1), namely the Brassicaceae (i.e., *Arabidopsis*, *Capsella*, *Brassica*, *Thellungiella*), legumes (*Glycine*, *Phaseolus*, *Cicer*, *Medicago* and *Lotus*), Malvaceae (*Theobroma* and *Gossypium*), *Linum* (flax), and *Fragaria* (strawberry). These results suggest a greater tendency of Type I SUTs to be duplicated and retained since their split from Type III, and this trend was particularly escalated in several lineages.

The Brassicaceae Type I members clustered into three strongly supported subclades, referred to as B1, B2, and B3 (Figure 2C). The well-characterized AtSUC2 (At1g22710), known to be involved in phloem loading (Truernit and Sauer, 1995; Gottwald et al., 2000; Srivastava et al., 2008), belongs to the small B1 clade that is basal to the expanded B2 and B3. All species were represented by a single *SUT* in the B1 clade, except *Brassica* that has experienced genome triplication. Orthologs in the B1 clade share high sequence similarities with one another (>94%), as well as conserved gene structure with the other angiosperm Type I members (Figure 3). The B2 clade contains two branches, the larger of which was similar to the B1 clade with mostly single-copy representation, including the pollen-expressing AtSUC1 (At1g71880), whereas the smaller branch contains Camelineae-specific (*Arabidopsis* and *Capsella*) tandem duplicates. The B3 clade is the largest, with 2–4 copies per species. Members of the B2 and B3 clades experienced an intron-loss event unlike the other angiosperm Type I *SUTs* (Figure 3). Synteny analysis based on the Plant Genome Duplication Database (Lee et al., 2012) showed that AtSUC2 (clade B1) and AtSUC1 (B2, main branch) reside in large collinear blocks that are also syntenic with many other species, whereas members of the B2 small branch and B3 clade showed no syntenic relationship outside of Brassicaceae. The B1 clade thus represents the founding member of the ancestral Brassicaceae genome. Based on *K*_s_ analysis (Figure 2E), we infer that the most recent common ancestor (MRCA) of clades B2 and B3 arose from B1 via Brassicaceae-specific *alpha* duplication (Bowers et al., 2003), whereas clade B3 likely originated from B2 via segmental duplications.

Similar to the Brassicaceae, the Type I SUT expansion in flax also involved multiple rounds of duplication as well as gene structure changes, including one divergent intronless member (Lus10007702, Supplemental Figure 1) that did not cluster with the other flax SUTs in the current phylogenetic tree (Figure 2A). The expansion scenario in the legumes (Fabeceae) is somewhat different, with all members retaining the conserved gene structure of angiosperm Type I *SUTs* (Figure 3). The legume SUTs formed three subclades (F1 to F3, Figure 2D), each with one or two members from the basal *Lotus*, suggesting their origin in the ancestral legume. Clade F1 appeared to be the founding group, with mostly single-copy members (except *Lotus*) that are highly similar to each other (87–97%). Based on *K*_s_ analysis (Figure 2E), the MRCA of clades F2 and F3 likely evolved from clade F1 via the legume-specific whole genome duplication (WGD) event (Schmutz et al., 2010). The F2 and F3 clades likely originated from a tandem duplication event in the ancestral legume shortly after the legume-specific WGD, since members in these two clades are located in tandem in both *Glycine* (soybean) and *Phaseolus* (common bean). Clade F3 members appear to be lost in Hologalegina (*Cicer* and *Medicago*) after their divergence from Millettioid (*Glycine* and *Phaseolus*). In some cases, additional lineage-specific duplications were observed (Figure 2D).

### AG2 was preferentially expanded in monocots, with evidence of positive selection

**Figure 4:**
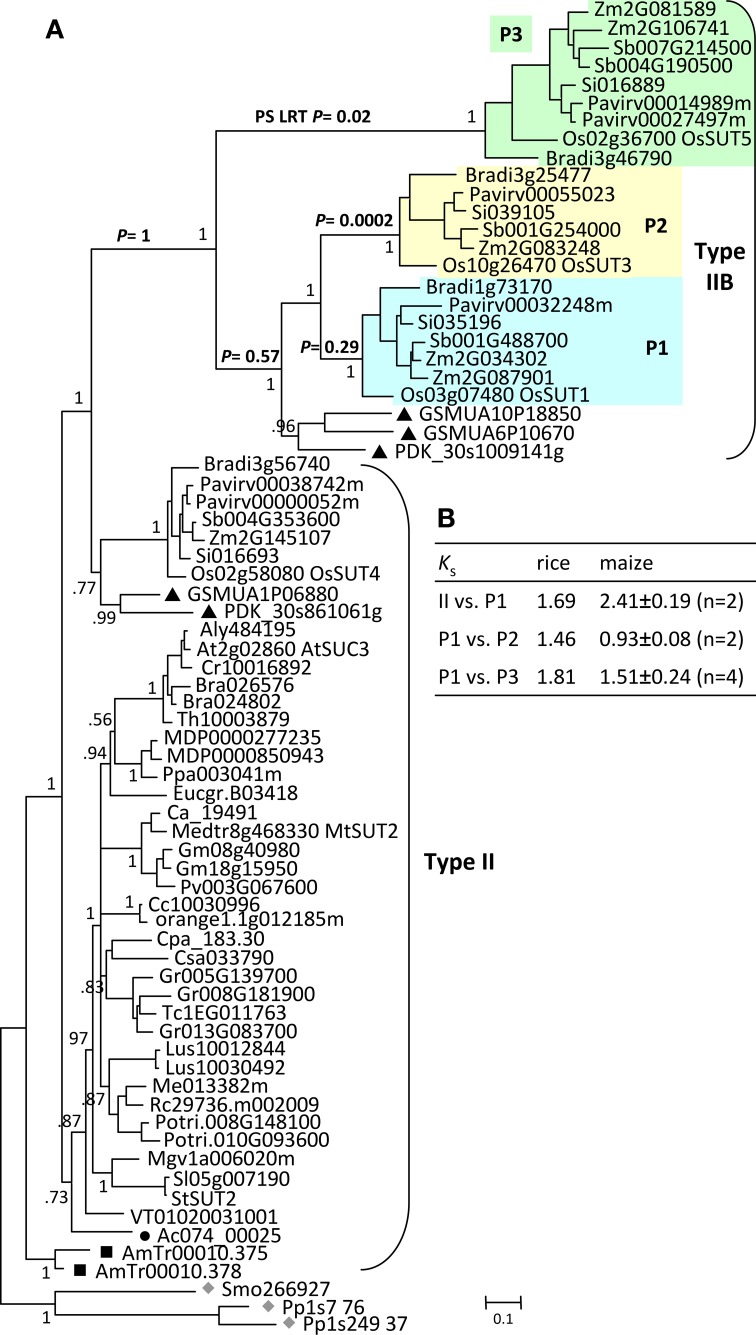
**Evolution of ancient group AG2 SUTs**. **(A)** Bayesian phylogenetic inference of AG2 SUTs. *Physcomitrella* and *Selaginella* members (gray diamonds) are basal to all other angiosperm SUTs. *Amborella* SUTs (black squares) are sister to modern angiosperm members that form eudicot- and monocot-specific branches, with *Aquilegia* (black circle) and *Phoenix*/*Musa* (black triangles) SUTs at the respective basal positions. Type IIB is derived from the monocot branch of Type II, forming three subclades P1, P2, and P3. The *P* values from the positive selection (PS) log likelihood ratio test (LRT) are shown for the major Type IIB branches. Posterior probabilities for major nodes are shown. Sequence names are provided in Supplemental Table 1. **(B)** *K*_s_ estimation between members of the expanded Type II/IIB SUTs in rice and maize. Values for maize are means and standard deviations of all gene pairs within each comparison group.

Like in AG1, moss and spikemoss SUTs were basal to angiosperm SUTs in the AG2 tree (Figure 4A). Two tandem copies of *Amborella* SUTs were basal to monocot and eudicot SUTs that formed two separate branches. The monocot- and eudicot-specific clustering is notably different from the separation of Type II (Group 3, monocots and eudicots) and Type IIB (Group 1, monocots only) in previous studies (Sauer, 2007; Braun and Slewinski, 2009; Payyavula et al., 2011; Doidy et al., 2012; Reinders et al., 2012). However, the pattern is consistent with the interpretation that all monocot SUTs within AG2 descended from one MRCA, sister to the eudicot MRCA. Two sub-clades were observed within the monocot branch, each accompanied by SUT members from basal monocots date palm and banana (Figure 4A). The smaller clade includes members that were previously classified as the monocot branch of the Type II/Group 3 (Figure 4A) (Sauer, 2007; Braun and Slewinski, 2009; Payyavula et al., 2011; Reinders et al., 2012). Their inferred evolutionary distance (branch length) was similar to that of the eudicot Type II members, with conserved gene structure that is also shared by the progenitor *Amborella* and spikemoss SUTs (Figure 3). The larger clade corresponds to the monocot-specific Type IIB (Group I in Sauer, 2007), with much longer branch lengths. Type IIB *SUTs* exhibit highly variable gene structures, not conserved with Type II (Figure 3). Together, these data suggest that Type IIB arose from Type II early in monocot evolution, before the divergence of Poales (grasses), Arecales (palm) and Zingiberales (banana).

The Type IIB group was further expanded in grasses after their divergence from basal monocots, forming three subclades (P1, P2, and P3, Figure 4A) each represented by all six sequenced grasses (*Oryza*, *Zea*, *Sorghum*, *Setaria, Pancium*, and *Brachypodium*). A similar observation was reported previously, with clade P3 corresponding to the monocot-specific Group 5 designated by Braun and Slewinski (2009), or the SUT5 clade described in Kühn and Grof (2010). Interestingly, Type IIB and its three subclades all descended from their MRCAs via long branches (Figure 4A). While this may be an artifact of long branch attraction arising from limited taxon sampling (Stefanovic et al., 2004), long branches were not observed in the monocot clades of Type II (or Type III) with the same taxon coverage. This led us to suspect a rapid evolution of Type IIB SUTs. We examined the non-synonymous (dN) and synonymous (dS) substitution rates of Type IIB as well as its sister Type II members across a sliding window of 100 codons (Supplemental Figure 2). Both SUT groups showed predominantly higher dS than dN, though some regions of Type II sequences exhibited a slightly elevated dN/dS ratio. The results suggested that Type II and Type IIB SUTs are under similar levels of purifying selection, likely due to functional constraints of coding sequences. This is consistent with the short branch length within each subclade. We next tested for positive selection that might have acted on the long branches leading to the three subclades in Type IIB. Results indicated that the branches leading to clades P2 (*p* = 0.0002) and P3 (*p* = 0.02), but not P1 (*p* = 0.29), were indeed under positive selection (Figure 4A). Six positively selected sites in P2 and P3 members were identified by BEB analysis with a probability of >0.95, and all six mapped to an 85 amino acids region near the C-terminus. No evidence of positive selection was detected for the branches leading to the MRCA of Type IIB (*p* = 1), or to the MRCA of clades P1, P2 and basal monocots (*p* = 0.57). Positive selection might have contributed to the numerous intron-loss events, and hence hypervariable gene structures observed for P2 and P3 genes (Figure 3). Together with *K*_s_ analysis (Figure 4B), we infer that P1 is the founding clade of Type IIB, with clades P2 and P3 undergoing positive selection following their origin from the pancereal *rho* and *sigma* duplications (Tang et al., 2010), respectively.

### Divergent expression of the expanded SUT groups in monocots and eudicots

**Figure 5:**
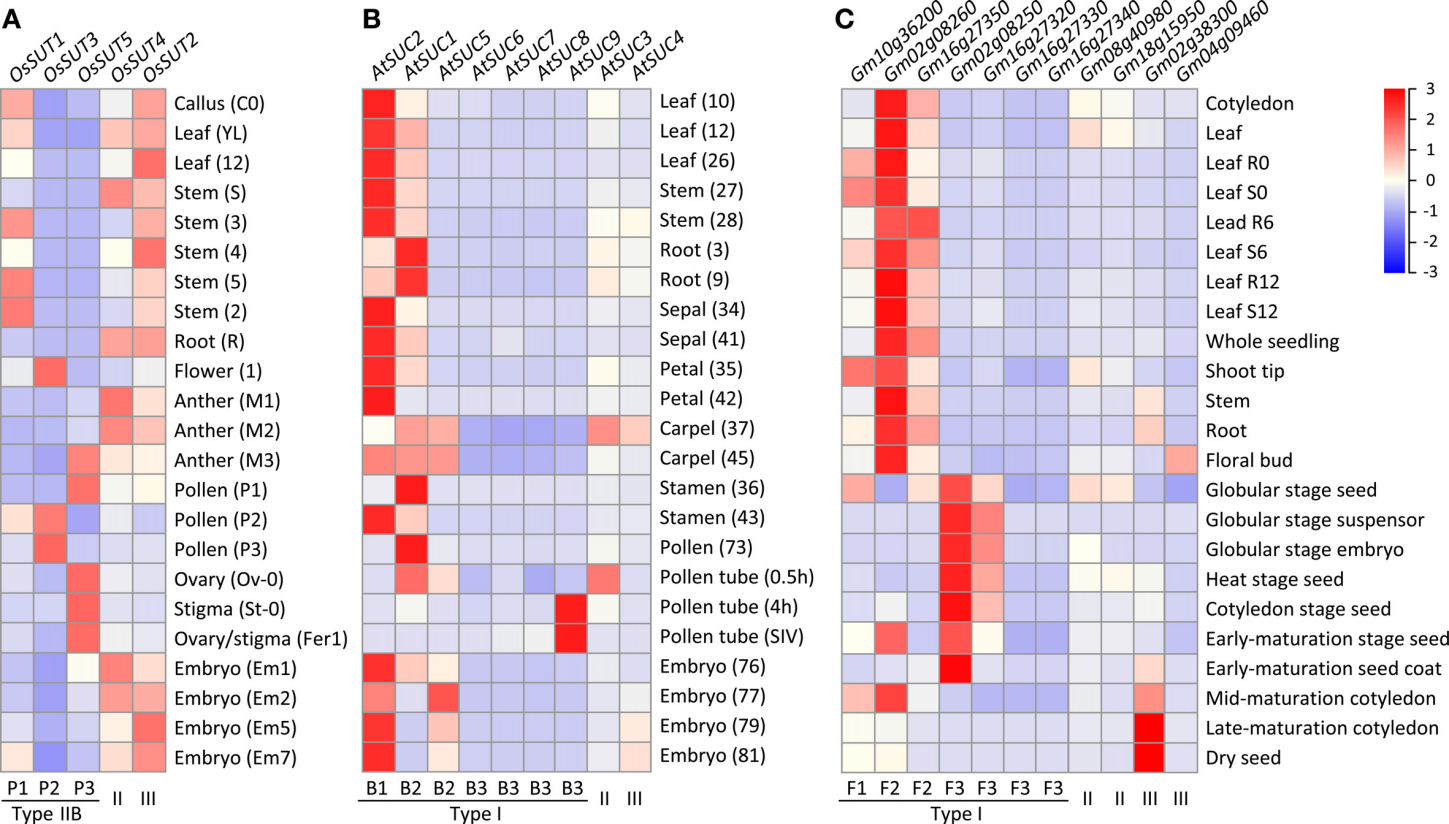
**Heatmap illustration of *SUT* gene expression profiles across vegetative and reproductive tissues**. **(A)** Rice *SUT* expression profiles. **(B)** *Arabidopsis SUT* gene expression profiles. **(C)** soybean *SUT* gene expression profiles. The scale bar depicts the relative expression strength (red, high expression; blue, low expression). SUT group and subclade designation are shown at the bottom. Tissue codes shown in parentheses for **(A,B)** are from the original data sources (see Materials and Methods).

To investigate the potential for functional diversification in the independently expanded monocot (Type IIB) and eudicot (Type I) SUT families, we examined their expression profiles by mining publicly available transcriptome data from diverse tissues of rice, *Arabidopsis thaliana* and soybean. In rice (Fujita et al., 2010; Kudo et al., 2013), the more ancestral Type II *OsSUT4* (Os02g58080) and Type III *OsSUT2* (Os12g44380) showed universal expression in all tissues analyzed (Figure 5A). Among the Type IIB *SUTs*, transcript levels of the founding member *OsSUT1* (Os03g07480, clade P1) were highest in vegetative tissues, especially in stems. This is consistent with previous reports of *OsSUT1* localization to phloem companion cells and sieve elements, and with its proposed functions in phloem loading and in carbohydrate storage and remobilization (Scofield et al., 2002, 2007a,b). *OsSUT1* transcripts were also present in reproductive tissues, but at lower levels. In contrast, transcripts of the positively selected members *OsSUT3* (Os10g26470, clade P2) and *OsSUT5* (Os02g36700, clade P3) were only detected in reproductive tissues, with spatiotemporal specificities (Figure 5A). During pollen development, *OsSUT5* transcripts were most abundant in uninucleate microspores, but declined sharply in subsequent (bi- and tri-cellular) stages. A reverse pattern was observed for *OsSUT3*, with highest levels found in tri-cellular pollen grains (Figure 5A). High levels of *OsSUT5* transcripts were also detected in ovary and stigma during fertilization and embryogenesis (Figure 5A). Similar spatiotemporal expression patterns were observed in indica rice (Wang et al., 2010; Peng et al., 2012) (data not shown). The complementary expression patterns of clades P2 and P3 *SUTs* in reproductive tissues are consistent with subfunctionalization following their divergence from clade P1 by positive selection in monocots.

In *Arabidopsis thaliana*, the Type I founding member *AtSUC2* (At1g22710, clade B1), known to function in phloem loading (Truernit and Sauer, 1995; Gottwald et al., 2000), exhibited the highest *SUT* transcript levels in most of the tissues examined (Figure 5B). *AtSUC1* (At1g71880) from clade B2 showed complementary expression, especially in roots and pollen where *AtSUC2* transcript levels were low. However, *AtSUC1* can complement the growth defects of *atsuc2* mutants when expressed from the *AtSUC2* promoter (Wippel and Sauer, 2012), suggesting subfunctionalization via partitioned expression between duplicated genes. Complementary expression was also observed between B2 members, with *AtSUC5* (At1g71890, tandem duplicate of *AtSUC1*) exhibiting an embryo-specific expression, and between B1/B2 and B3 clades, with *AtSUC9* (At5g06170) transcripts detected at very high levels in germinating pollen tubes (Qin et al., 2009), followed by *AtSUC8* (At2g14670) and *AtSUC7* (At1g66570) (Figure 5B). *AtSUC7* and *AtSUC6* (At5g43610) are pseudogenes, exhibiting extensive alternative splicing (*AtSUC7*) or sequence substitutions (*AtSUC6*) that are predicted to encode aberrant proteins (Sauer et al., 2004). Together, our analysis showed that multiple rounds of duplication in the Brassicaceae gave rise to an expanded Type I subfamily, with evidence of subfunctionalization in reproductive tissues.

In soybean, the most broadly expressed *SUT* genes belong to Type I clades F1 (Glyma10g36200) and F2 (Glyma02g08260 and Glyma16g27350), and their transcript levels were generally higher in vegetative than reproductive tissues (Figure 5C). In contrast, two members of the expanded clade F3 (Glyma02g08250 and Glyma16g27320) were much more highly expressed in embryonic and seed tissues, complementary to the pattern of clade F2 members. Expression of the remaining two F3 members was near the detection limit (Figure 5C). Thus, similar to the findings from *Arabidopsis thaliana*, lineage-specific expansion of Type I SUTs in soybean was also followed by expression partitioning in reproductive tissues.

## Discussion

**Figure 6:**
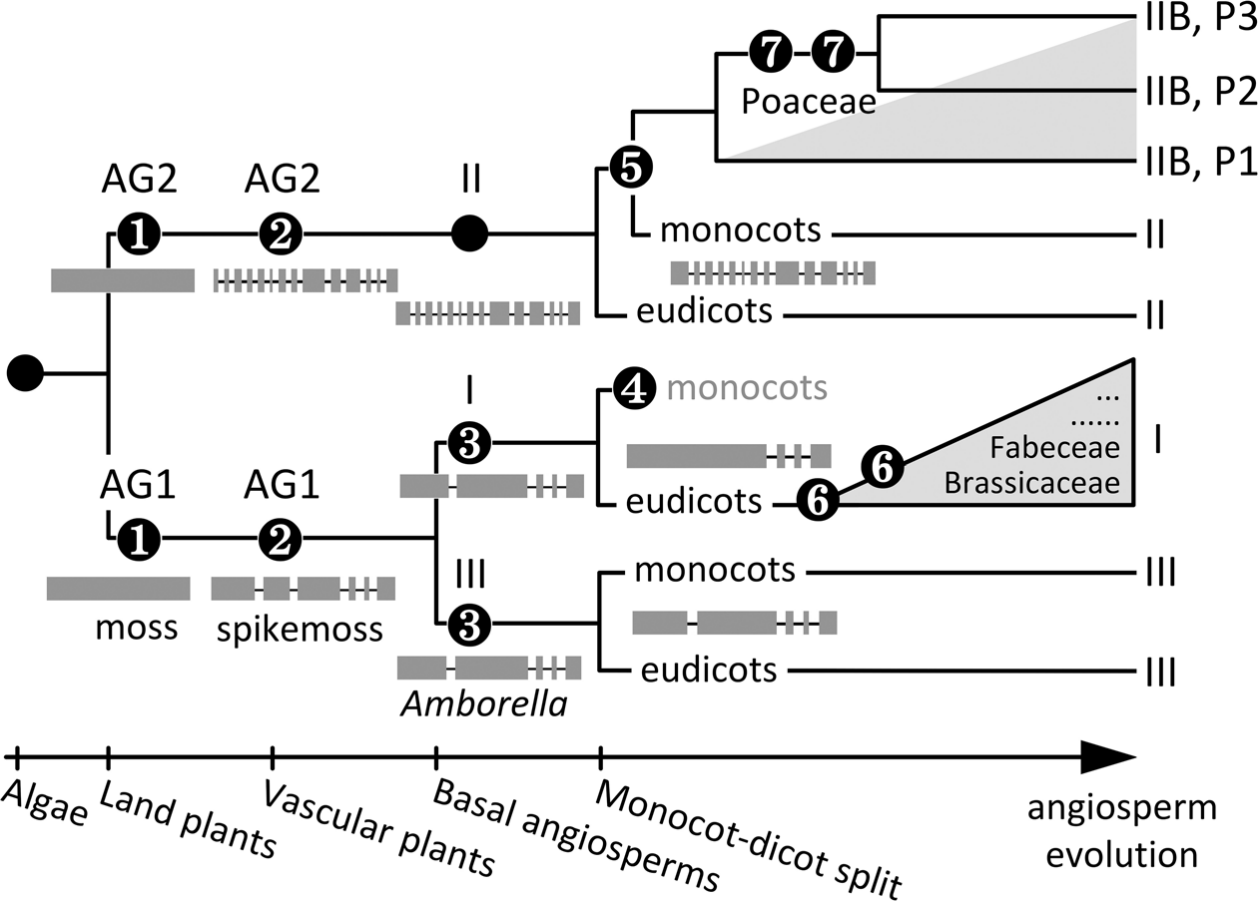
**Schematic representation of SUT family evolution in angiosperms**. Major events include (1) SUT divergence into AG1 and AG2 in the ancestral bryophyte, (2) intron acquisition of AG1 and AG2, and expansion and diversification of AG1 SUTs in the progenitor vascular plant, (3) diversification of Type I and Type III SUTs in the basal angiosperm, (4) loss of Type I SUTs in the ancestral monocot, (5) origin of Type IIB from Type II in the ancestral monocot, (6) intron loss and recurring episodes of lineage-specific Type I expansion (gray triangle)in eudicot‘ (7) multiple cereal-specific duplications of Type IIB (gray triangle) yielding three subclades (P1 to P3). Modern Type II and Type III SUTs represent evolutionarily conserved descendants of ancient groups AG2 and AG1, respectively. The reference landmarks of angiosperm evolution at the bottom are not to scale.

The Bayesian inference of SUT phylogeny from 41 genome-sequenced plant taxa, including six basal lineages, has uncovered a complex evolutionary history of both ancient and relatively recent origins. The presence of distinct AG1 and AG2 SUTs in moss (Event 1, Figure 6) suggests that *SUT* diverged very early in the ancestral bryophyte, perhaps concomitant with terrestrial colonization. Major adaptations, such as osmoregulation, desiccation tolerance, and acquisition of elaborate transport capabilities to support growth and carbon-based metabolism (Rensing et al., 2008) have all been associated with higher plant SUT functions (Sauer, 2007; Kühn and Grof, 2010; Aoki et al., 2012; Frost et al., 2012). Another major evolutionary event of ancient *SUTs* is the divergence of gene structure (i.e., acquisition of introns) dating back to the advent of vascular plants (Event 2, Figure 6): moss *SUTs* are intronless within the CDS, whereas spikemoss *SUTs* harbor 5 and 13 introns in AG1 and AG2, respectively, similar to modern angiosperm Type II and Type III members. Interestingly, the expanded AG1 SUTs are more divergent in spikemoss (71–79% amino acid sequence similarity with one another) than in moss (88–95% similarity). This suggests that diversification of AG1 SUT may be important for developmental innovations associated with the transition from non-vascular to vascular growth habits, namely the evolution of a dominant, vascularized and branched plant body with roots, shoots and leaves (Langdale, 2008). Variation in gene sequence and structure may be linked to functional adaptation of SUTs to cope with the increasing complexity of intra/intercellular distribution of sucrose in lycophytes. Diversification of AG1 SUTs ultimately led to distinct Type I and Type III SUTs in *Amborella* that share a 67% amino acid sequence similarity and the same intron loss event from the progenitor AG1 (Event 3, Figure 6). Type I SUT was subsequently lost in the monocot lineages (Event 4), perhaps concomitant with evolution of Type IIB from Type II in these taxa (Event 5, Figure 6). Lineage-specific expansion of Type I (Event 6) and Type IIB (Event 7) further shaped SUT family evolution in modern angiosperms.

Modern Type I and Type III SUTs are functionally distinct, with plasma membrane-localized Type I involved in apoplastic phloem loading and tonoplastic Type III modulating vacuolar sucrose efflux (Sauer, 2007; Kühn and Grof, 2010; Braun et al., 2014). It was previously proposed that Type I originated from Type III via loss of the vacuolar targeting sequence after monocots and dicots diverged (Reinders et al., 2012). While our analysis supports Type I as the derived group, its evolution from Type III predates the ancestral angiosperm (Event 3, Figure 6). Both inclusion of basal lineages and gene structure analysis were instrumental for clarifying this aspect of SUT family evolution. Diversification of Type I and Type III SUTs may be linked to evolution of phloem (i.e., ‘modern’ phloem with specialized accessory cells) to support long-distance transport of photoassimilates (van Bel, 1999). Type I SUT has contrasting fates in modern flowering plants: this group was lost in monocots (Event 4) but was significantly expanded in eudicots following an intron-loss event in the progenitor eudicot (Event 6). The presence of multiple Type I *SUTs* in the majority of eudicot taxa we examined, including both highly divergent (e.g., *Vitis* and *Mimulus*) and similar (e.g., *Populus* and *Malus*) paralogs (Figure 2A), suggested preferential and recurring retentions of Type I duplicates following ancient as well as recent WGDs. Indeed, lineage-specific duplication/retention is the most significant driver of Type I subfamily expansion. We observed expression partitioning of the expanded Type I *SUTs* in both *Arabidopsis* and soybean, with genes descending from the most recent duplication (i.e., clades B3 and F3, respectively) exhibiting reproductive tissue-specific expression. Thus, expansion and subfunctionalization of Type I *SUT* appear to be a recurring theme in some eudicot lineages, with adaptive significance in reproduction.

Like Type III of AG1, Type II represents the evolutionarily conserved subfamily in AG2, with predominately single-copy presence, conserved gene structure across monocots and eudicots, and a phylogeny largely congruent with the species tree. Type II differs from the other SUTs with an extended cytoplasmic loop and as-yet-undefined function, though they are localized to the plasma membrane (Sauer, 2007; Kühn and Grof, 2010; Braun et al., 2014). Type IIB likely arose from Type II via the monocot-specific ancient polyploidy event reported by Tang et al. (2010), followed by loss of the central cytoplasmic loop in the progenitor of commelinid monocots (Event 5, Figure 6). In a striking parallelism to eudicot Type I SUTs, Type IIB (founding clade P1) was significantly expanded in Poaceae, giving rise to clades P2 and P3 (Event 7). Both the P2 and P3 clades were under positive selection, which might have contributed to their functional specialization. As is the case with the expanded *Arabidopsis* and soybean Type I families, partitioned tissue expression is evident among the expanded rice Type IIB family, with duplicated members also exhibiting biased expression in reproductive tissues. These data support convergent evolution of the expanded Type I and Type IIB SUTs in multiple unrelated angiosperm taxa, whereby the more recently derived members independently acquired specialized expression in reproductive tissues.

Interestingly, functional similarity between independently evolved Type I and Type IIB SUTs has also been reported for their founding (more ancestral) members, e.g., clades B1 and P1, respectively. Several of these SUTs (e.g., *Arabidopsis* AtSUC2, rice OsSUT1 and maize ZmSUT1/Zm2G034302) are plasma membrane-localized and function in apoplastic phloem loading (Truernit and Sauer, 1995; Gottwald et al., 2000; Scofield et al., 2007a; Slewinski et al., 2009). Expression of the barley *HvSUT1* (ortholog of OsSUT1 and ZmSUT1) successfully complemented the growth defect of the *Arabidopsis atsuc2* mutant, supporting functional equivalence of Type I and Type IIB founding members (Reinders et al., 2012). Convergent evolution of phylogenetically distinct SUTs in phloem loading may be associated with independent vascular development of monocots and dicots during angiosperm evolution. Monocots and dicots differ in their vascular organization, and monocots lack vascular cambia to support secondary growth (Scarpella and Meijer, 2004). Because secondary growth predates the gymnosperm-angiosperm split, it is believed that the vascular cambium was lost in the ancestral monocot (Spicer and Groover, 2010). This is consistent with the more ancestral (pre-*Amborella*) origin of Type I SUT (Event 3, Figure 6), and their adaptive function in phloem loading accompanying evolution of phloem and secondary growth. The loss of the vascular cambium in monocots likely rendered Type I disposable (Event 4). Acquisition of phloem loading function by the expanded Type IIB SUT (Event 5) likely co-evolved with the highly modified vascular system of monocots. Convergent evolution of reproductive tissue-biased expression of Type I and Type IIB SUTs in distinct taxa is consistent with the dependence of reproductive sink organs on phloem-mediated long-distance transport of sucrose (Gottwald et al., 2000).

In summary, the present work expands on previous studies and identifies several key drivers of plant SUT family evolution. Marked gene structure and sequence divergence of AG1 SUT accompanied the early evolution of vascular plants, culminating in functionally distinct Type I and Type III SUT families predating basal angiosperms. The evolutionarily conserved Type II and Type III SUTs appear to be under purifying selection after recurring WGD events in angiosperm evolution, whereas Type I and Type IIB SUTs underwent differential evolution, via gene loss and/or expansion, in a lineage-specific manner. Independent episodes of convergent evolution of eudicot Type I and monocot Type IIB SUTs are linked to differential vascular development in these taxa, associated with SUT function in phloem loading and reproductive fitness of flowering plants. Our work also provides a phylogenomic basis for unifying the nomenclature of plant SUT family.

### Conflict of interest statement

The authors declare that the research was conducted in the absence of any commercial or financial relationships that could be construed as a potential conflict of interest.

## References

[B1] Al-Dous E. K. George B. Al-Mahmoud M. E. Al-Jaber M. Y. Wang H. Salameh Y. M.. (2011). *De novo* genome sequencing and comparative genomics of date palm (*Phoenix dactylifera*). Nat. Biotechnol. 29, 521–527. 10.1038/nbt.1860 21623354

[B2] Amborella Genome Project. (2013). The Amborella genome and the evolution of flowering plants. Science 342:1241089. 10.1126/science.1241089 24357323

[B3] Aoki N. Hirose T. Furbank R. (2012). Sucrose transport in higher plants: from source to sink, in Photosynthesis, eds Eaton-Rye J. J. Tripathy B. C. Sharkey T. D. (Netherlands: Springer), 703–729 10.1007/978-94-007-1579-0_28

[B4] Aoki N. Hirose T. Scofield G. N. Whitfeld P. R. Furbank R. T. (2003). The sucrose transporter gene family in rice. Plant Cell Physiol. 44, 223–232. 10.1093/pcp/pcg030 12668768

[B5] Ayre B. G. (2011). Membrane-transport systems for sucrose in relation to whole-plant carbon partitioning. Mol. Plant 4, 377–394. 10.1093/mp/ssr014 21502663

[B6] Barker L. Kühn C. Weise A. Schulz A. Gebhardt C. Hirner B.. (2000). SUT2, a putative sucrose sensor in sieve elements. Plant Cell 12, 1153–1164. 10.1105/tpc.12.7.1153 10899981 PMC149056

[B7] Barth I. Meyer S. Sauer N. (2003). PmSUC3: characterization of a SUT2/SUC3-type sucrose transporter from Plantago major. Plant Cell 15, 1375–1385. 10.1105/tpc.010967 12782730 PMC156373

[B8] Bowers J. E. Chapman B. A. Rong J. Paterson A. H. (2003). Unravelling angiosperm genome evolution by phylogenetic analysis of chromosomal duplication events. Nature 422, 433–438. 10.1038/nature01521 12660784

[B9] Braun D. M. Slewinski T. L. (2009). Genetic control of carbon partitioning in grasses: roles of sucrose transporters and tie-dyed loci in phloem loading. Plant Physiol. 149, 71–81. 10.1104/pp.108.129049 19126697 PMC2613709

[B10] Braun D. M. Wang L. Ruan Y.-L. (2014). Understanding and manipulating sucrose phloem loading, unloading, metabolism, and signalling to enhance crop yield and food security. J. Exp. Bot. 65, 1713–1735. 10.1093/jxb/ert416 24347463

[B11] Burge S. W. Daub J. Eberhardt R. Tate J. Barquist L. Nawrocki E. P.. (2013). Rfam 11.0: 10 years of RNA families. Nucleic Acids Res. 41, D226–D232. 10.1093/nar/gks1005 23125362 PMC3531072

[B12] Chang S. Wang Y. Lu J. Gai J. Li J. Chu P.. (2013). The mitochondrial genome of soybean reveals complex genome structures and gene evolution at intercellular and phylogenetic levels. PLoS ONE 8:e56502. 10.1371/journal.pone.0056502 23431381 PMC3576410

[B13] Cheadle C. Vawter M. P. Freed W. J. Becker K. G. (2003). Analysis of microarray data using Z score transformation. J. Mol. Diagn. 5, 73–81. 10.1016/S1525-1578(10)60455-2 12707371 PMC1907322

[B14] Chincinska I. Gier K. Krügel U. Liesche J. He H. Grimm B.. (2013). Photoperiodic regulation of the sucrose transporter StSUT4 affects the expression of circadian-regulated genes and ethylene production. Front. Plant Sci. 4:26. 10.3389/fpls.2013.00026 23429841 PMC3576705

[B15] Cox M. Peterson D. Biggs P. (2010). SolexaQA: at-a-glance quality assessment of Illumina second-generation sequencing data. BMC Bioinformatics 11:485. 10.1186/1471-2105-11-485 20875133 PMC2956736

[B16] Decourteix M. Alves G. Brunel N. Ameglio T. Guilliot A. Lemoine R.. (2006). JrSUT1, a putative xylem sucrose transporter, could mediate sucrose influx into xylem parenchyma cells and be up-regulated by freeze-thaw cycles over the autumn-winter period in walnut tree (*Juglans regia L.*). Plant Cell Environ. 29, 36–47. 10.1111/j.1365-3040.2005.01398.x 17086751

[B17] Dereeper A. Guignon V. Blanc G. Audic S. Buffet S. Chevenet F.. (2008). Phylogeny.fr: robust phylogenetic analysis for the non-specialist. Nucleic Acids Res. 36, W465–W469. 10.1093/nar/gkn180 18424797 PMC2447785

[B18] D'Hont A. Denoeud F. Aury J.-M. Baurens F.-C. Carreel F. Garsmeur O.. (2012). The banana (*Musa acuminata*) genome and the evolution of monocotyledonous plants. Nature 488, 213–217. 10.1038/nature11241 22801500

[B19] Doidy J. van Tuinen D. Lamotte O. Corneillat M. Alcaraz G. R. Wipf D. (2012). The *Medicago truncatula* sucrose transporter family: characterization and implication of key members in carbon partitioning towards arbuscular mycorrhizal fungi. Mol. Plant 5, 1346–1358. 10.1093/mp/sss079 22930732

[B20] Eom J. S. Cho J. I. Reinders A. Lee S. W. Yoo Y. Tuan P. Q.. (2011). Impaired function of the tonoplast-localized sucrose transporter in rice, OsSUT2, limits the transport of vacuolar reserve sucrose and affects plant growth. Plant Physiol. 157, 109–119. 10.1104/pp.111.176982 21771914 PMC3165862

[B21] Flemetakis E. Dimou M. Cotzur D. Efrose R. C. Aivalakis G. Colebatch G.. (2003). A sucrose transporter, LjSUT4, is up-regulated during *Lotus japonicus* nodule development. J. Exp. Bot. 54, 1789–1791. 10.1093/jxb/erg179 12754265

[B22] Frost C. J. Nyamdari B. Tsai C.-J. Harding S. A. (2012). The tonoplast-localized sucrose transporter in Populus (PtaSUT4) regulates whole-plant water relations, responses to water stress, and photosynthesis. PLoS ONE 7:e44467. 10.1371/journal.pone.0044467 22952983 PMC3432113

[B23] Fujita M. Horiuchi Y. Ueda Y. Mizuta Y. Kubo T. Yano K.. (2010). Rice expression atlas in reproductive development. Plant Cell Physiol. 51, 2060–2081. 10.1093/pcp/pcq165 21062870

[B24] Gautier L. Cope L. Bolstad B. M. Irizarry R. A. (2004). affy—analysis of Affymetrix GeneChip data at the probe level. Bioinformatics 20, 307–315. 10.1093/bioinformatics/btg405 14960456

[B25] Gottwald J. R. Krysan P. J. Young J. C. Evert R. F. Sussman M. R. (2000). Genetic evidence for the in planta role of phloem-specific plasma membrane sucrose transporters. Proc. Natl. Acad. Sci. U.S.A. 97, 13979–13984. 10.1073/pnas.250473797 11087840 PMC17686

[B26] Hackel A. Schauer N. Carrari F. Fernie A. R. Grimm B. Kühn C. (2006). Sucrose transporter LeSUT1 and LeSUT2 inhibition affects tomato fruit development in different ways. Plant J. 45, 180–192. 10.1111/j.1365-313X.2005.02572.x 16367963

[B27] Holder M. Lewis P. O. (2003). Phylogeny estimation: traditional and Bayesian approaches. Nat. Rev. Genet. 4, 275–284. 10.1038/nrg1044 12671658

[B28] Hunt M. Kaur N. Stromvik M. Vodkin L. (2011). Transcript profiling reveals expression differences in wild-type and glabrous soybean lines. BMC Plant Biol. 11:145. 10.1186/1471-2229-11-145 22029708 PMC3217893

[B29] Jin J. Liu J. Wang H. Wong L. Chua N.-H. (2013). PLncDB: plant long non-coding RNA database. Bioinformatics 29, 1068–1071. 10.1093/bioinformatics/btt107 23476021 PMC3624813

[B30] Katoh K. Standley D. M. (2013). MAFFT multiple sequence alignment software version 7: improvements in performance and usability. Mol. Biol. Evol. 30, 772–780. 10.1093/molbev/mst010 23329690 PMC3603318

[B31] Kim K. H. Kang Y. J. Kim D. H. Yoon M. Y. Moon J.-K. Kim M. Y.. (2011). RNA-Seq analysis of a soybean near-isogenic line carrying bacterial leaf pustule-resistant and -susceptible alleles. DNA Res. 18, 483–497. 10.1093/dnares/dsr033 21987089 PMC3223079

[B32] Kramer E. M. (2009). Aquilegia: a new model for plant development, ecology, and evolution. Annu. Rev. Plant Biol. 60, 261–277. 10.1146/annurev.arplant.043008.092051 19575583

[B33] Kudo T. Akiyama K. Kojima M. Makita N. Sakurai T. Sakakibara H. (2013). UniVIO: a multiple omics database with hormonome and transcriptome data from rice. Plant Cell Physiol. 54, e9. 10.1093/pcp/pct003 23314752 PMC3583028

[B34] Kühn C. (2003). A comparison of the sucrose transporter systems of different plant species. Plant Biol. 5, 215–232 10.1055/s-2003-40798

[B35] Kühn C. Grof C. P. L. (2010). Sucrose transporters of higher plants. Curr. Opin. Plant Biol. 13, 287–297. 10.1016/j.pbi.2010.02.001 20303321

[B36] Lalonde S. Frommer W. B. (2012). SUT sucrose and MST monosaccharide transporter inventory of the Selaginella genome. Front. Plant Sci. 3:24. 10.3389/fpls.2012.00024 22645575 PMC3355790

[B37] Lalonde S. Wipf D. Frommer W. B. (2004). Transport mechanisms for organic forms of carbon and nitrogen between source and sink. Annu. Rev. Plant Biol. 55, 341–372. 10.1146/annurev.arplant.55.031903.141758 15377224

[B38] Langdale J. A. (2008). Evolution of developmental mechanisms in plants. Curr. Opin. Genet. Dev. 18, 368–373. 10.1016/j.gde.2008.05.003 18573341

[B39] Lee T.-H. Tang H. Wang X. Paterson A. H. (2012). PGDD: a database of gene and genome duplication in plants. Nucleic Acids Res. 41, D1152–D1158. 10.1093/nar/gks1104 23180799 PMC3531184

[B40] Lemoine R. Bürkle L. Barker L. Sakr S. Kühn C. Regnacq M.. (1999). Identification of a pollen-specific sucrose transporter-like protein NtSUT3 from tobacco. FEBS Lett. 454, 325–330. 10.1016/S0014-5793(99)00843-1 10431832

[B41] Meyer S. Lauterbach C. Niedermeier M. Barth I. Sjolund R. D. Sauer N. (2004). Wounding enhances expression of AtSUC3, a sucrose transporter from Arabidopsis sieve elements and sink tissues. Plant Physiol. 134, 684–693. 10.1104/pp.103.033399 14739351 PMC344544

[B42] Meyer S. Melzer M. Truernit E. Hümmer C. Besenbeck R. Stadler R.. (2000). AtSUC3, a gene encoding a new Arabidopsis sucrose transporter, is expressed in cells adjacent to the vascular tissue and in a carpel cell layer. Plant J. 24, 869–882. 10.1046/j.1365-313x.2000.00934.x 11135120

[B43] Miller M. A. Pfeiffer W. Schwartz T. (2010). Creating the CIPRES Science Gateway for inference of large phylogenetic trees, in Proceedings of the Gateway Computing Environments Workshop (GCE), New Orleans B2 - Proceedings of the Gateway Computing Environments Workshop (GCE), New Orleans. (New York, NY: Institute of Electrical and Electronics Engineers).

[B44] Payyavula R. S. Tay K. H. C. Tsai C.-J. Harding S. A. (2011). The sucrose transporter family in Populus: the importance of a tonoplast PtaSUT4 to biomass and carbon partitioning. Plant J. 65, 757–770. 10.1111/j.1365-313X.2010.04463.x 21261761

[B45] Peng H. Chun J. Ai T.-B. Tong Y.-A. Zhang R. Zhao M.-M.. (2012). MicroRNA profiles and their control of male gametophyte development in rice. Plant Mol. Biol. 80, 85–102. 10.1007/s11103-012-9898-x 22403030

[B46] Qin Y. Leydon A. R. Manziello A. Pandey R. Mount D. Denic S.. (2009). Penetration of the stigma and style elicits a novel transcriptome in pollen tubes, pointing to genes critical for growth in a pistil. PLoS Genet. 5:e1000621. 10.1371/journal.pgen.1000621 19714218 PMC2726614

[B47] Quast C. Pruesse E. Yilmaz P. Gerken J. Schweer T. Yarza P.. (2013). The SILVA ribosomal RNA gene database project: improved data processing and web-based tools. Nucleic Acids Res. 41, D590–D596. 10.1093/nar/gks1219 23193283 PMC3531112

[B48] Ranwez V. Harispe S. Delsuc F. Douzery E. J. P. (2011). MACSE: multiple alignment of coding sequences accounting for frameshifts and stop codons. PLoS ONE 6:e22594. 10.1371/journal.pone.0022594 21949676 PMC3174933

[B49] Reinders A. Sivitz A. B. Ward J. M. (2012). Evolution of plant sucrose uptake transporters (SUTs). Front. Plant Sci. 3:22. 10.3389/fpls.2012.00022 22639641 PMC3355574

[B50] Rensing S. A. Lang D. Zimmer A. D. Terry A. Salamov A. Shapiro H.. (2008). The Physcomitrella genome reveals evolutionary insights into the conquest of land by plants. Science 319, 64–69. 10.1126/science.1150646 18079367

[B51] Riesmeier J. W. Hirner B. Frommer W. B. (1993). Potato sucrose transporter expression in minor veins indicates a role in phloem loading. Plant Cell 5, 1591–1598. 10.1105/tpc.5.11.1591 8312741 PMC160388

[B52] Ronquist F. Teslenko M. van der Mark P. Ayres D. L. Darling A. Höhna S.. (2012). MrBayes 3.2: efficient Bayesian phylogenetic inference and model choice across a large model space. Syst. Biol. 61, 539–542. 10.1093/sysbio/sys029 22357727 PMC3329765

[B53] Saski C. Lee S.-B. Daniell H. Wood T. Tomkins J. Kim H.-G.. (2005). Complete chloroplast genome sequence of Glycine max and comparative analyses with other legume genomes. Plant Mol. Biol. 59, 309–322. 10.1007/s11103-005-8882-0 16247559

[B54] Sauer N. (2007). Molecular physiology of higher plant sucrose transporters. FEBS Lett. 581, 2309–2317. 10.1016/j.febslet.2007.03.048 17434165

[B55] Sauer N. Ludwig A. Knoblauch A. Rothe P. Gahrtz M. Klebl F. (2004). AtSUC8 and AtSUC9 encode functional sucrose transporters, but the closely related AtSUC6 and AtSUC7 genes encode aberrant proteins in different Arabidopsis ecotypes. Plant J. 40, 120–130. 10.1111/j.1365-313X.2004.02196.x 15361146

[B56] Scarpella E. Meijer A. H. (2004). Pattern formation in the vascular system of monocot and dicot plant species. New Phytol. 164, 209–242 10.1111/j.1469-8137.2004.01191.x 33873557

[B57] Schmid M. Davison T. S. Henz S. R. Pape U. J. Demar M. Vingron M.. (2005). A gene expression map of *Arabidopsis thaliana* development. Nat. Genet. 37, 501–506. 10.1038/ng1543 15806101

[B58] Schmutz J. Cannon S. B. Schlueter J. Ma J. Mitros T. Nelson W.. (2010). Genome sequence of the palaeopolyploid soybean. Nature 463, 178–183. 10.1038/nature08670 20075913

[B59] Schneider S. Hulpke S. Schulz A. Yaron I. Holl J. Imlau A.. (2012). Vacuoles release sucrose via tonoplast-localised SUC4-type transporters. Plant Biol. 14, 325–336. 10.1111/j.1438-8677.2011.00506.x 21972845

[B60] Scofield G. N. Aoki N. Hirose T. Takano M. Jenkins C. L. D. Furbank R. T. (2007a). The role of the sucrose transporter, OsSUT1, in germination and early seedling growth and development of rice plants. J. Exp. Bot. 58, 483–495. 10.1093/jxb/erl217 17138625

[B61] Scofield G. N. Hirose T. Aoki N. Furbank R. T. (2007b). Involvement of the sucrose transporter, OsSUT1, in the long-distance pathway for assimilate transport in rice. J. Exp. Bot. 58, 3155–3169. 10.1093/jxb/erm153 17728297

[B62] Scofield G. N. Hirose T. Gaudron J. A. Furbank R. T. Upadhyaya N. M. Ohsugi R. (2002). Antisense suppression of the rice transporter gene, OsSUT1, leads to impaired grain filling and germination but does not affect photosynthesis. Funct. Plant Biol. 29, 815–826 10.1071/PP01204 32689529

[B63] Shiratake K. (2007). Genetics of sucrose transporters. Genes Genomes Genomics 1, 73–80.

[B64] Slewinski T. L. Meeley R. Braun D. M. (2009). Sucrose transporter1 functions in phloem loading in maize leaves. J. Exp. Bot. 60, 881–892. 10.1093/jxb/ern335 19181865 PMC2652052

[B65] Spicer R. Groover A. (2010). Evolution of development of vascular cambia and secondary growth. New Phytol. 186, 577–592. 10.1111/j.1469-8137.2010.03236.x 20522166

[B66] Srivastava A. C. Ganesan S. Ismail I. O. Ayre B. G. (2008). Functional characterization of the Arabidopsis AtSUC2 sucrose/H+ symporter by tissue-specific complementation reveals an essential role in phloem loading but not in long-distance transport. Plant Physiol. 148, 200–211. 10.1104/pp.108.124776 18650401 PMC2528097

[B67] Stadler R. Brandner J. Schulz A. Gahrtz M. Sauer N. (1995). Phloem loading by the PmSUC2 sucrose carrier from Plantago major occurs into companion cells. Plant Cell 7, 1545–1554. 10.1105/tpc.7.10.1545 12242355 PMC161007

[B68] Stadler R. Truernit E. Gahrtz M. Sauer N. (1999). The AtSUC1 sucrose carrier may represent the osmotic driving force for anther dehiscence and pollen tube growth in Arabidopsis. Plant J. 19, 269–278. 10.1046/j.1365-313X.1999.00527.x 10476074

[B69] Stefanovic S. Rice D. Palmer J. (2004). Long branch attraction, taxon sampling, and the earliest angiosperms: Amborella or monocots? BMC Evol. Biol. 4:35. 10.1186/1471-2148-4-35 15453916 PMC543456

[B70] Tamura K. Peterson D. Peterson N. Stecher G. Nei M. Kumar S. (2011). MEGA5: molecular evolutionary genetics analysis using maximum likelihood, evolutionary distance, and maximum parsimony methods. Mol. Biol. Evol. 28, 2731–2739. 10.1093/molbev/msr121 21546353 PMC3203626

[B71] Tang H. Bowers J. E. Wang X. Paterson A. H. (2010). Angiosperm genome comparisons reveal early polyploidy in the monocot lineage. Proc. Natl. Acad. Sci. 107, 472–477. 10.1073/pnas.0908007107 19966307 PMC2806719

[B72] Trapnell C. Roberts A. Goff L. Pertea G. Kim D. Kelley D. R.. (2012). Differential gene and transcript expression analysis of RNA-seq experiments with TopHat and Cufflinks. Nat. Protoc. 7, 562–578. 10.1038/nprot.2012.016 22383036 PMC3334321

[B73] Truernit E. Sauer N. (1995). The promoter of the *Arabidopsis thaliana* SUC2 sucrose-H+ symporter gene directs expression of beta-glucuronidase to the phloem: evidence for phloem loading and unloading by SUC2. Planta 196, 564–570. 10.1007/BF00203657 7647685

[B74] van Bel A. J. E. (1999). Evolution, polymorphology and multifunctionality of the phloem system. Perspect. Plant Ecol. Evol. Syst. 2, 163–184 10.1078/1433-8319-00069

[B75] Wang L. Xie W. Chen Y. Tang W. Yang J. Ye R.. (2010). A dynamic gene expression atlas covering the entire life cycle of rice. Plant J. 61, 752–766. 10.1111/j.1365-313X.2009.04100.x 20003165

[B76] Wippel K. Sauer N. (2012). Arabidopsis SUC1 loads the phloem in suc2 mutants when expressed from the SUC2 promoter. J. Exp. Bot. 63, 669–679. 10.1093/jxb/err255 22021573 PMC3254675

[B77] Yang Z. (2007). PAML 4: Phylogenetic analysis by maximum likelihood. Mol. Biol. Evol. 24, 1586–1591. 10.1093/molbev/msm088 17483113

[B78] Yang Z. Wong W. S. Nielsen R. (2005). Bayes empirical bayes inference of amino acid sites under positive selection. Mol. Biol. Evol. 22, 1107–1118. 10.1093/molbev/msi097 15689528

[B79] Zhang J. Nielsen R. Yang Z. (2005). Evaluation of an improved branch-site likelihood method for detecting positive selection at the molecular level. Mol. Biol. Evol. 22, 2472–2479. 10.1093/molbev/msi237 16107592

[B80] Zhang Z. Li J. Zhao X. Q. Wang J. Wong G. K. Yu J. (2006). KaKs_Calculator: calculating Ka and Ks through model selection and model averaging. Genomics Proteomics Bioinformatics 4, 259–263. 10.1016/S1672-0229(07)60007-2 17531802 PMC5054075

[B81] Zhou Y. C. Qu H. X. Dibley K. E. Offler C. E. Patrick J. W. (2007). A suite of sucrose transporters expressed in coats of developing legume seeds includes novel pH-independent facilitators. Plant J. 49, 750–764. 10.1111/j.1365-313X.2006.03000.x 17253986

