# Supplementary material for: Bayesian phylogeny of sucrose transporters: ancient origins, differential expansion and convergent evolution in monocots and dicots
Source: Front Plant Sci. 2014 Nov 12;5:615. doi: 10.3389/fpls.2014.00615 (PMC4228843; doi:10.3389/fpls.2014.00615)
Supplement: Supplementary file 2 [file Image1.PDF]

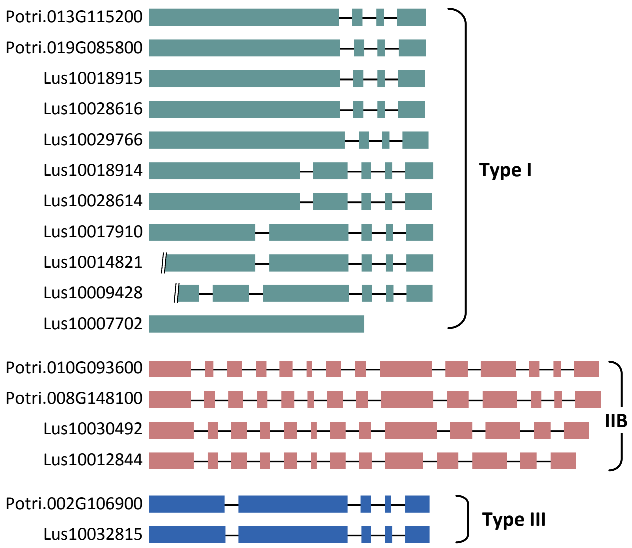

Supplemental Figure 1. Exon-intron structure of SUT genes from flax (*Linum usitatissimum*). Genes are color-coded by phylogenetic group (note, two Type I gene models contain sequence gaps within the first exon). *Populus* (Potri) SUTs with conserved gene structures are included as references. Introns are not drawn to scale.
