# Supplementary material for: Bayesian phylogeny of sucrose transporters: ancient origins, differential expansion and convergent evolution in monocots and dicots
Source: Front Plant Sci. 2014 Nov 12;5:615. doi: 10.3389/fpls.2014.00615 (PMC4228843; doi:10.3389/fpls.2014.00615)
Supplement: Supplementary file 3 [file Image2.PDF]

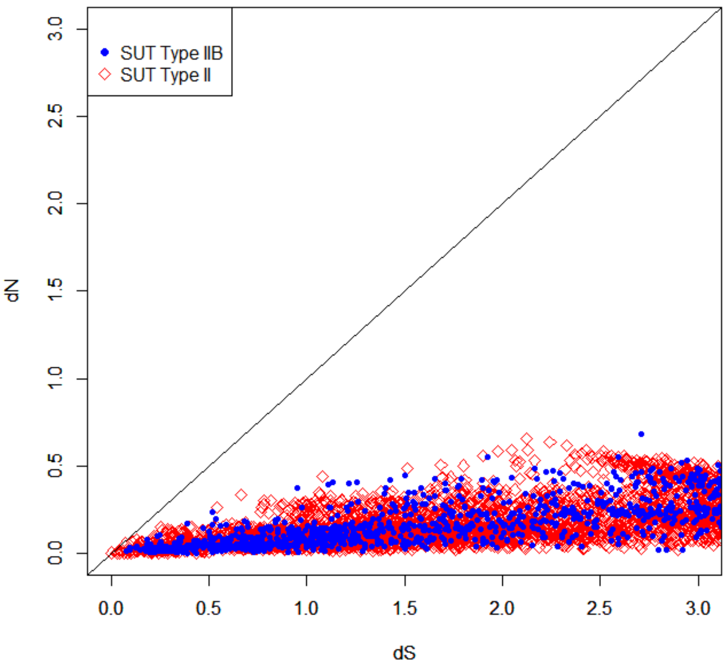

Supplemental Figure 2. Plot of dN versus dS of Type II and Type IIB SUTs from monocots. Similar levels of purifying selection were detected, despite the long branches leading to Type IIB members. Each data point represents a 100-codon window from the alignment. The diagonal line denotes neutral selection (dN=dS).
